# Protein homeostasis in the aged and diseased heart

**DOI:** 10.20517/jca.2023.4

**Published:** 2023-03-07

**Authors:** Nirjal Mainali, Srinivas Ayyadevara, Akshatha Ganne, Robert J. Shmookler Reis, Jawahar L. Mehta

**Affiliations:** 1 Bioinformatics Program, University of Arkansas at Little Rock and University of Arkansas for Medical Sciences, Little Rock, AR 72205, USA.; 2 Department of Geriatrics and Institute on Aging, University of Arkansas for Medical Sciences, Little Rock, AR 72205, USA.; 3 Central Arkansas Veterans Healthcare Service, Little Rock, AR 72205, USA.; 4 Division of Cardiology, University of Arkansas for Medical Sciences and Central Arkansas Veterans Healthcare System, Little Rock, AR 72205, USA.

**Keywords:** Protein aggregation, cardiovascular disease, aging, myocardial ischemia, hypertension

## Abstract

Protein homeostasis, the balance between protein synthesis and degradation, requires the clearance of misfolded and aggregated proteins and is therefore considered to be an essential aspect of establishing a physiologically effective proteome. Aging alters this balance, termed “proteostasis”, resulting in the progressive accumulation of misfolded and aggregated proteins. Defective proteostasis leads to the functional deterioration of diverse regulatory processes during aging and is implicated in the etiology of multiple pathological conditions underlying a variety of neurodegenerative diseases and in age-dependent cardiovascular disease. Detergent-insoluble protein aggregates have been reported by us in both aged and hypertensive hearts. The protein constituents were found to overlap with protein aggregates seen in neurodegenerative diseases such as Alzheimer’s disease. Therefore, targeting these protein components of aggregates may be a promising therapeutic strategy for cardiovascular pathologies associated with aging, ischemia, and/or hypertension.

## Graphical Abstract

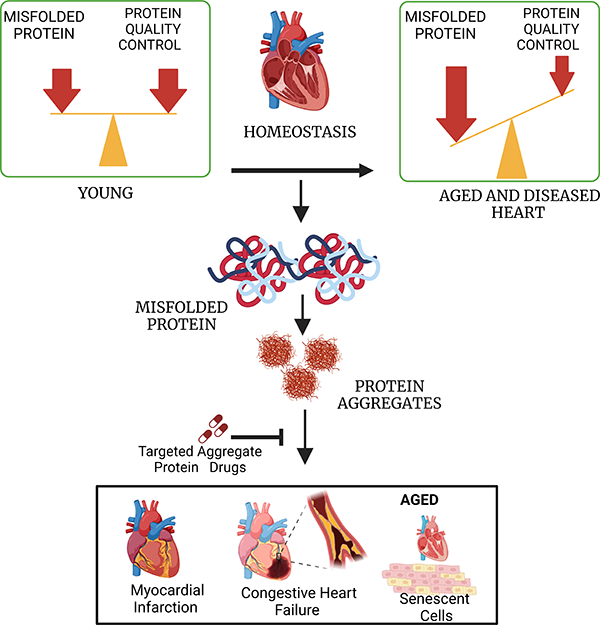

## INTRODUCTION

Cardiovascular diseases (CVD) have been and remain the leading cause of death worldwide, and account for 23.5% of U.S. mortality (CDC 2017). Hypertension, high blood cholesterol, diabetes, obesity, smoking, physical inactivity, gender, age, and genetics are the leading contributors to CVDs. Disruption of protein homeostasis, accumulation of misfolded proteins, and protein aggregation have also been linked to several types of CVDs^[1]^. A deeper understanding of protein homeostasis and protein aggregation, and their impairment in diseased hearts, may enable the development of new strategies and therapeutic targets for CVD prevention and intervention.

Protein homeostasis is fundamental for proper cell function and survival. A healthy proteome contains both proteins that are properly folded into a well-structured native state, as well as many intrinsically disordered proteins which have regions lacking a stable structure, or which achieve stability only upon binding of one or more ligands, or in association with other proteins in dimeric or multimeric complexes. Whereas most proteins fold spontaneously, many proteins require molecular chaperones to assist in folding^[2,3]^. There are several monitoring systems in the cell that detect any proteins that have been misfolded, partially folded, or are intrinsically disordered or unstructured. Misfolded proteins have exposed hydrophobic regions that can adhere to similar regions in nearby proteins, leading to nonfunctional binding and aggregation. Accumulating such aberrant complexes was previously shown to be highly toxic to cells^[4]^.

The accumulation of misfolded proteins in a cell or subcellular compartment elicits the Unfolded Protein Response (UPR), which recruits chaperones required for proper protein folding^[5–8]^. Accumulation of misfolded proteins in the endoplasmic reticulum (ER) triggers the ER-associated degradation (ERAD) pathway via glucose-regulated protein 78 (GRP78), inositol-requiring protein 1 (IRE1), activating transcription factor 6 (ATF-6), and PKR-like ER kinase (PERK). The UPR pathway helps to limit misfolding by halting or reducing protein synthesis at the endoplasmic reticulum^[9–12]^. The Ubiquitin-Proteasome System (UPS) chiefly handles misfolded proteins, whereas autophagy provides an alternative degradation route for degraded organelles and larger aggregates^[13–16]^.

## THE UBIQUITIN-PROTEASOME SYSTEM

UPS is a highly regulated process that degrades proteins, most of which are structurally aberrant (and thus potentially dysfunctional), due to misfolding, oxidation, excessive post-translational misfolding, and/or entanglement of intrinsically disordered proteins or regions. UPS machinery has been identified in nucleus and cytosol, and degrades proteins from all cell compartments including membranes, nucleus, cytoplasm, and the ER lumen. UPS is an ATP-dependent system comprising two complementary processes: ubiquitination and proteasomal degradation^[17,18]^. Ubiquitination occurs in three steps. First, a ubiquitin-activating enzyme (E1) binds ATP and transfers AMP-adenylate to a C-terminal carboxylate; it then captures that ubiquitin monomer through a Cys-thioester linkage and transfers it to carrier protein E2 via a thioester exchange. Ubiquitin ligases (E3) then catalyze substrate-specific ubiquitination of target proteins. There are over 600 substrate-specific E3 ligases^[19]^, which recognize misfolded target proteins (or their associated chaperones) and catalyze the transfer of the ubiquitin moiety to proteins destined to be degraded after assembly of a polyubiquitin chain. Since ubiquitin modification is a dynamic process, not all polyubiquitinated proteins are destined for degradation; they also serve as signaling molecules that trigger DNA repair or activate kinases and stabilize certain proteins in cancer cells^[20–22]^. Polyubiquitinated proteins destined to be degraded are transferred to proteasomes, where they are deubiquitinated and cleaved into fragments of < 30 residues^[23,24]^.

UPS in the mammalian heart is distinguished by several features. E3 ligases such as atrogin-1, the muscle ring finger (MuRF) family, and C-terminal HSP70-interacting protein (CHIP) assist in the conjugation of ubiquitin to substrates peculiar to or especially abundant in the heart^[25]^. All proteasomes consist of two sub-complexes: a 20S proteasome, also known as the catalytic core particle (CP), and one or two 19S proteasome activators, also known as regulatory particles (RPs). The CP comprises subpopulations of α and β catalytic subunits, which exist in constitutive forms (β1, β2, and β5), as well as immune forms (β1i, β2i, and β5i) that make up the immunoproteasome^[26,27]^. The 20S core of cardiac proteasomes contains a mixture of constitutive and immune proteasome subunits. The 19S regulatory particle may also be replaced by an 11S regulatory particle in mouse heart, complexed with the 20S catalytic core particle. The resulting 20S-11S complex appears to improve the catabolism of many cardiac substrates^[17,28,29]^.

## UPS IN THE DISEASED HEART

The relationship between abnormal UPS activity and pathogenesis was first recognized in diverse neurodegenerative diseases, including Alzheimer’s, Parkinson’s, and Huntington’s diseases. Subsequently, several studies have determined the role of UPS and UPS-related therapeutic targets in disease states of other organs, including the heart^[30,31]^. MDM2 (an E3 ligase) has been shown to target the p53 protein, which is both a tumor suppressor and an inducer of apoptosis, for ubiquitination and proteasomal processing^[32,33]^. Elevated levels of p53 and MDM2 have been observed in heart failure patients, suggesting that increased apoptosis may be directly related to UPS dysregulation in diseased hearts^[34]^. Several studies have reported that proteasome subunits can undergo post-translational modifications (PTM) or oxidative damage that inhibit their catabolic function in ischemia-reperfusion (IR) injury models, thus perhaps contributing to UPS dysfunction leading to pathologic hypertrophy during and after CVD^[35–37]^. Total ubiquitinated protein load has been found to increase in several cardiovascular pathologies^[38–41]^. Impairment in coupling of ubiquitination and proteasomal degradation has also been shown to be a contributing factor to models of myocardial ischemic-reperfusion injury in both yeast and mice^[42,43]^.

## AUTOPHAGY

Lysosomes are the key degradative organelles in autophagy, an intracellular “recycling” pathway that serves to eliminate large, defective cellular components including cell organelles (e.g., mitochondria) as well as pathogens and cytosolic aggregates. Such structures are too large to be degraded by proteasomes but can be cytotoxic if not eliminated. Recycling of damaged and/or harmful cellular components by degradation allows the recovery of ATP, amino acids, and nucleotides for cell metabolism. There are three main types of autophagy: macroautophagy, microautophagy, and chaperone-mediated autophagy (CMA). Previous studies have also discovered crosstalk between UPS and autophagy^[44]^. Under normal conditions or moderate stress, autophagy clears old and damaged cell organelles, and dysfunctional protein complexes, as a cytoprotective alternative to apoptosis. The balance between apoptosis and autophagy, essential for proper functioning of the heart, is maintained by mTOR and Endoplasmic Reticulum (ER) stress pathways but disrupted by rapamycin treatment^[45]^. Cardiomyocytes, specialized muscle cells that maintain the contraction-relaxation cycle of the heart, are rich in mitochondria - resulting in a dependence on mitochondrial autophagy (termed “mitophagy”) for achieving homeostasis.

## AUTOPHAGY IN THE DISEASED HEART

Mortality of cardiac cells can arise via apoptosis, ischemic cell death, or autophagic cell death^[46–49]^. Mice with *Atg5-* or *Atg7*-deficient hearts are more susceptible to cardiac dysfunction induced by ER and mitochondrial stress than mice with wild-type hearts^[50,51]^. Cardiomyocytes in hearts from animal models of ischemia-reperfusion (I/R) injury have elevated abundance of autophagosomes, suggesting an impairment in their clearance after I/R injury^[52]^. Mice with myocardial infarction (MI) were found to have reduced autophagic flux and mitochondrial respiration relative to sham-operated mice. This decrease in flux results in an accumulation of damaged organelles, polyubiquitinated proteins, and spheroidal (more rounded) mitochondria^[53,54]^. Autophagy is an essential protective mechanism that fails during long-term heart dysfunction^[49,55–57]^. Oxidative damage to proteins required for autophagic functions (e.g., Atg4, Atg5, and Atg7) can lead to the failure of cardioprotection in CVD^[58–61]^.

## CROSSTALK AND COOPERATION BETWEEN UPS AND AUTOPHAGY

Inhibition of UPS proteasomes disrupts the ERAD pathway and increases ER stress markers (e.g., IRE1, ATF6, PERK). IRE1 activation results in recruitment of TRAF2 required for phosphorylation of JNK, and expression of autophagy-pathway genes. Upon cleavage, ATF6 translocates to the nucleus and triggers expression of DAPK1 and phosphorylation of beclin-1 required for autophagosome biogenesis. Also, activated PERK through eIF2alpha activates ATF4 and CHOP, in turn inducing expression of diverse autophagy (ATG) genes^[62–66]^. Inhibition of autophagy leads to compromised UPS-associated substrate protein clearance^[67]^. Shuttling proteins, such as SQSTM-1/p62, responsible for the proper conveyance of ubiquitinated substrates to the proteasome, also accumulate in aggregates due to the inhibition of autophagy^[67,68]^.

UPS and autophagy also work together in the clearance of unnatural, misfolded, and unrequired proteins^[26,69]^. This has been well documented in Huntington’s disease, Parkinson’s disease, and amyotrophic lateral sclerosis (ALS), characterized by aggregation of their respective hallmark proteins such as Htt. α-synuclein and TDP-43. All these aggregates require both proteasomal and lysosomal degradation pathways^[70,71]^. Also, molecular chaperones such as CHIP and BAG have multiple functionalities. CHIP acts as a co-chaperone for HSP70 and HSP90 for proteasomal degradation, but can also mediate autophagic degradation of Lys63-specific misfolded proteins^[72,73]^. Among proteins in the BAG family, BAG1 mediates proteasomal degradation, while BAG3 cooperates with Hsp70, CHIP and SQSTM-1/p62 for autophagic degradation^[74,75]^.

## MOLECULAR MECHANISMS LEADING TO CVD

Myocardial ischemia and heart failure (HF) are the most common conditions that lead to CVD mortality. Each of these conditions initiates hypoxia, oxidative stress, ER and mitochondrial stress, and cardiac remodeling. Several studies have shown that a cascade of cellular-stress events can alter the machinery of UPS and/or autophagy, resulting in their disrupted clearance functions and the intracellular accumulation of misfolded proteins^[38,69,75–79].^

## HYPOXIA (ISCHEMIA)

Organ-specific oxygen consumption depends on the type and condition of each tissue. The heart and brain create the highest demand for oxygen, and cardiac consumption rises sharply during vigorous exercise. Hypoxia refers to an oxygen level (partial pressure) that is below the physiological requirements of an organism or a tissue. Genes that are elicited in hypoxic environments are termed hypoxia-inducible and are upregulated by hypoxia-inducible factor-1α (HIF-1α) and HIF-2α acting as transcription factors or co-factors. In normoxic conditions, HIF-1α is hydroxylated through an oxygen-dependent process and is degraded through a UPS pathway. HIF-1α protein is stabilized in hypoxic conditions and translocates into the nucleus, where it binds to hypoxia-response elements (HREs) to activate the transcription of hypoxia-inducible genes. Genes directly targeted by HIF-1α are chiefly those responsible for glycolysis, angiogenesis, and erythropoiesis, but there are numerous secondary targets^[80–82]^. Hypoxia negatively impacts protein synthesis through such mechanisms as inhibition of mTOR signaling, and activation of PERK in the ER - both of which are responsible for regulation of translation initiation^[83–86]^. Hypoxia also leads to activation of UPR machinery. Hypoxia-induced phosphorylation of eIF2α activates ATF4, a transcription factor that regulates autophagy and expression of CHOP (which induces apoptosis) and GADD34 (involved in the integrated stress response pathway)^[87,88]^. Hypoxia also induces protein misfolding, especially in the ER, which is itself a trigger of inflammation pathways^[88]^. Disrupted UPR, an oxygen-dependent process, contributes to the accumulation of misfolded proteins in aggregates. Other branches of the ER-stress pathway, mediated by ATF6 and IRE1, are also activated by hypoxia^[89,90]^. Additional pathways impacted by hypoxia include apoptosis, cell-cycle regulation, DNA repair, and metabolism, as shown by microarray analysis comparing normoxic *vs.* hypoxic cells^[91]^.

Decreased oxygen delivery, leading to tissue hypoxia, occurs in several cardiovascular diseases including atherosclerosis, hypertension, and heart failure. In atherosclerosis, monocytes recruited by inflammatory signals to the arterial wall differentiate into macrophages, whose nitric oxide (NO) production is enhanced by HIF-1α but opposed by HIF-2α^[92,93]^. Since NO is a primary mediator of inflammation, hypoxia is pro-inflammatory in atherosclerosis. HIF-1α has been shown to regulate hypertension in mice; HIF-1α^+/−^ mice show a significant decrease in hypertension induced by chronic-intermittent hypoxia compared to wild-type mice^[94,95]^. In humans, chronic hypoxia causes activation of the sympathetic nervous system and increased systemic arterial pressure^[96]^. Prolonged hypoxia leads to activation of autophagy, which protects cardiomyocytes from ER stress and apoptosis^[97,98]^. Angiotensin receptor-neprilysin inhibitor (ARNI), a compound recently developed to treat heart failure, reduces hypoxia-induced myocardial injury through inhibition of autophagy^[99]^.

## OXIDATIVE STRESS

Elevated levels of free radicals, chiefly reactive oxygen species (ROS), result in the disruption of several cell-signaling and growth-mediating pathways. At low physiological levels, ROS play a critical role in diverse cellular processes including phosphorylation and activation of several transcription factors^[100,101]^. Protein carbonylation increases during oxidative stress, as observed for the 19S regulatory S6 proteasome subunit, considered to be the oxidation-sensitive subunit in 26S proteasomes^[102]^. Post-translational modifications related to oxidative stress, such as carbonylation, were shown to accumulate in proteins in aged mice^[103]^. Carbonylation of side-chain amino acids is linked to protein aggregation by promoting unfolding^[104,105]^.

ROS are generated as metabolic by-products of oxidative phosphorylation by mitochondria, which are especially abundant in cardiomyocytes; however, they also originate from nitric oxide synthase (NOS) and diverse other enzymes such as NADPH oxidase (NOX) and cytochromes p450. Mitochondria play a dominant role in determining ROS levels, as they amplify NOX-derived ROS and thus function as redox hubs in cardiac physiology and disease. High ROS levels affect the disposition of myocardial calcium, induce arrhythmia, and can contribute to cardiac remodeling^[106]^. In addition to free radicals generated in the myocardium (cardiomyocytes), leukocytes produce a large proportion of cardiac ROS. Although small amounts of ROS are also generated under normal physiological conditions, the antioxidant defense mechanisms are often adequate to remove excess ROS and maintain oxidative balance. However, during hypoxia, the oxidant load surpasses the heart’s ability to counter the ROS load resulting in oxidative stress. ROS are highly reactive toward proteins, lipids, and DNA; oxidative damage to DNA leads to somatic mutations, the vast majority of which are deleterious, whereas their impact on proteins and membranes can result in cell dysfunction or death. Alterations in protein and calcium homeostasis due to oxidative stress have been previously implicated in the heart, where they are most readily observed through structural modifications to cardiomyocytes^[107,108]^. NOX4, a member of NADPH oxidase family of proteins, is responsible for transfer of electrons from either NADPH or NADH to molecular oxygen to generate O_2_^−[109,110]^. NOX4 is constitutively active, and elevation of its expression is closely associated with increased oxidative stress^[111]^. Mitochondrial abundance of NOX4 is upregulated in aging heart leading to increased ROS production and cardiac hypertrophy^[112,113]^. HSP17, one of the heat shock proteins, was shown to ameliorate myocardial injury and oxidative stress^[114–117]^.

## ER STRESS

The endoplasmic reticulum (ER) is a vital organelle responsible for essential cellular functions such as protein synthesis, protein folding, calcium homeostasis, and the generation of autophagosomes. Under normal physiological conditions, GRP78 (a key ER chaperone) remains bound to effector molecules such as ATF6, IRE1, and PERK; during stress, however, these effectors dissociate from GRP78 and become activated. Activated ATF6 is cleaved in Golgi bodies, releasing its N-terminal fragment that acts as a transcription factor to induce genes that protect against ER stress and ROS. Activated IRE1 splices mRNA encoding X-box binding protein 1 (*XBP1*); the spliced *XBP1* isoform translocates to the nucleus to initiate transcription of molecular chaperones. Activated PERK phosphorylates eukaryotic initiation factor 2 alpha (eIF2α) which in turn activates ATF4 and then C/EBP homologous protein (CHOP)^[118]^. As discussed above, there is interplay and crosstalk between various pathways involved in ER stress, hypoxia, and UPS. Senstrin2 is an important regulator of cell responses to various stresses^[119,120]^. In a recent study, ER stress was shown to induce cardiac autophagy and myocardial dysfunction in Sestrin2-knockout mice^[121]^.

Mild ER stress is beneficial for the cell, as it clears any unfolded or damaged proteins via an ER-specific UPR termed UPR^ER^; however, prolonged ER stress leads to cell death and is cardiotoxic since it leads to apoptosis of cardiomyocytes^[122]^. Because cardiomyocytes cannot regenerate, extended periods of ER stress can cause irreparable damage to the heart. Since ER plays a vital role in the metabolism of lipids and the maturation and folding of transmembrane proteins, suppression of ER stress is critical to survival. ER stress nevertheless occurs in atherosclerotic plaques in humans and in animal models of atherosclerosis, although ameliorated by flavonoids^[123–125]^.

## MITOCHONDRIAL STRESS

Mitochondria are another essential cell organelle responsible for generating ATP as the principal energy currency required for cell metabolism and enabling cardiomyocyte contraction and relaxation. Other critical functions of mitochondria include biosynthetic metabolic processes, chiefly through the Krebs cycle, the maintenance of sustainable ROS levels, monitoring protein integrity, and preventing the accumulation of damaged proteins^[118]^. Under physiological stress, mitochondria activate their own UPR, termed UPR^Mit^. This initiates transcription of the mitochondrial protease ClpP, mitochondrial chaperones or heat-shock proteins (HSP60 and HSP10), and proteins involved in ROS detoxification. The transcription of these factors is believed to be initiated by activated ATF5. Another stress-induced pathway is the activation of PERK and c-Jun N-terminal kinase 2 (JNK2), both of which participate in the translation of ATF4, CHOP, and ATF5 - proteins that contribute to cardiomyocyte dysfunction and apoptotic cell death. Mitochondrial stress also activates an autophagy-variant clearance pathway to remove dysfunctional mitochondria, called mitophagy^[126,127]^. Glutathione is involved in cellular homeostasis, protects cells from oxidative stress, and inhibits apoptosis^[128]^. Treatment with exogenous glutathione can help to restore mitochondrial redox status and eventually decrease mitochondrial stress leading to improvement in cardiovascular function of aged mice^[129]^.

Crosstalk between mitochondria and ER has been observed and is believed to be a vital process in maintaining cardiac function. Calcium sequestered in ER is channeled to mitochondria, where it is required for ATP production. Proteins implicated in mitochondrial calcium uptake include Mfn2, VAPB, PTPIP51, and STIM1. STIM1 is an ER-resident calcium sensor; its genetic knockout in mice disrupts both ER and mitochondrial functions, leading to cardiac fibrosis and myopathy. Similar interactions between ER and mitochondria are implicated by autophagy dysregulation via JNK, Bcl-2, and Atg6^[130–132]^. Both mitochondrial and ER stress responses are tightly connected to other mechanisms involved in protein homeostasis, including UPS, autophagy, hypoxia, and oxidative stress during cardiac dysfunction; consequently, proteins involved in these stress responses might be valuable targets for therapeutic interventions.

## CARDIAC REMODELING

Cardiac tissue is susceptible to damage after ischemic injury, or cardiomyopathy, arising from hypoxia, oxidative stress, ER, or mitochondrial stress. Changes in size, mass, geometry, and function of the heart, as well as its overall morphology and function, are observed after cardiac injury - changes collectively known as cardiac remodeling. Following ischemic injury, there is extensive death of cardiomyocytes. The necrotic tissue after MI is replaced by extracellular matrix (ECM), resulting in fibrosis. Cardiac fibroblasts have high plasticity and are involved in the generation of ECM, scar formation, and inter-cellular signaling^[133–135]^. They increase their collagen synthesis, causing fibrosis not only of infarct tissue but also of non-infarct areas, thus participating in further cardiac remodeling^[136,137]^. Although initial remodeling of the heart is important to compensate for damage incurred, prolonged remodeling leads to the deterioration of cardiac function and may culminate in heart failure^[138]^. Prolonged remodeling of the heart is associated with ventricular arrhythmias, increased neurohormonal activation, and excessive vasoconstriction^[139,140]^. Several treatment strategies to reverse cardiac remodeling after heart failure have been studied in recent years. Renin-angiotensin system modulators, beta-blockers, neprilysin inhibitors and aldosterone antagonists are some of those drugs shown to promote angiogenesis after MI and to alter cardiac remodeling^[141,142]^.

## RELATIONSHIP BETWEEN MYOCARDIAL ISCHEMIA, AGING, AND HYPERTENSION

Hypertension, a common consequence of vascular remodeling, is a major health problem that often accompanies aging. With increasing age, the arteries and blood vessels become stiffer, which increases vascular resistance. Elevated blood pressure leads to CVD, kidney disease, vascular dementia, and/or eye problems. We recently reported that detergent-insoluble protein aggregates, isolated from aging and hypertensive mouse hearts, increased several-fold relative to young, normal hearts^[143]^. About 50% of all aggregate proteins increased in abundance with aging, while 55% increased with hypertension; a shared 30% of aggregate proteins increased with both aging and hypertension^[143]^. Remarkably, one-fifth of these “overlap” aggregate proteins were previously implicated in age-related neurodegenerative diseases (e.g., Alzheimer’s disease) and CVDs^[143–147]^. Many of these proteins are involved in protein homeostasis and specifically in UPS, including proteasome α subunits, and NEDD4, an E3 ligase that increases in protein aggregates with aging and hypertension^[143,148]^. Similarly, TOMM22 and TOMM40, proteins involved in mitochondrial protein import, are enriched in hypertensive mouse hearts^[143,149]^. NADH dehydrogenase implicated in cardiotoxicity through oxidative stress is seen to be enriched in both aged and hypertensive mice hearts. Other proteins such as Serine peptidase inhibitor 1 (Serpinh1) and Prohibitin are enriched with aging and hypertension^[143,150]^.

**Figure 1.**
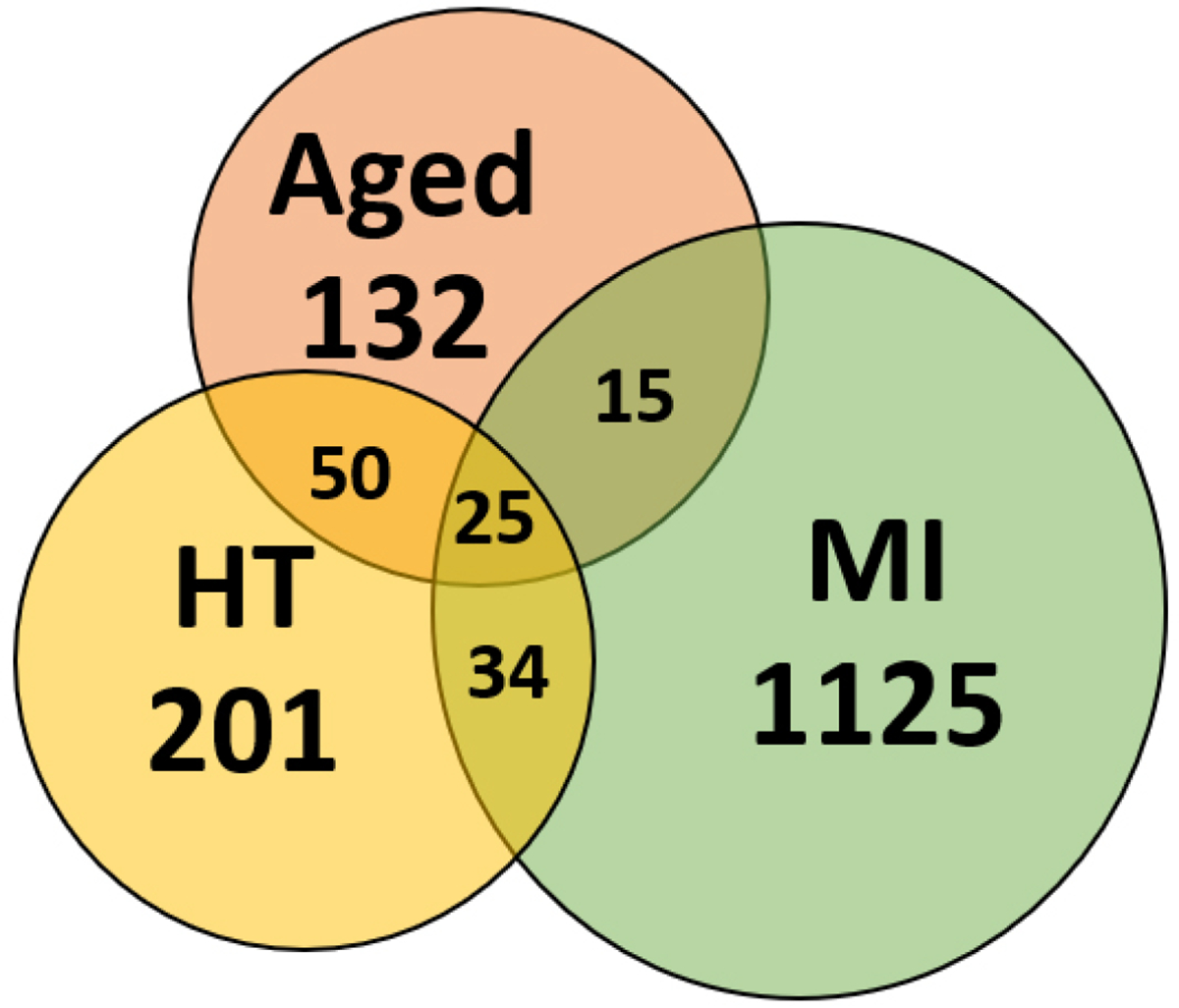
Proteins sequestered into aggregates overlap between aged, hypertensive (HT), and infarcted (MI) hearts.

Myocardial infarction (MI) following prolonged ischemia is associated with cardiomyocyte death (necrosis or apoptosis)^[151]^. Post-MI cardiomyocyte death is followed by cardiac remodeling (heart chamber dilation, ventricular wall thinning, and loss of cardiac function). Several studies have reported that following MI, cardiomyocytes undergo an unfolded protein response (UPR) that leads to increased apoptosis^[152]^. Prolonged ischemia results in mitochondrial swelling and the release of cytochrome C, contributing to contractile dysfunction^[153]^. In the ER, ischemia leads to impaired protein folding due to abnormal activation and dysregulation of ER chaperones, contributing to protein misfolding, impaired calcium homeostasis, and apoptosis^[154]^. After simulating MI in wild-type mice with left coronary artery (LCA) ligation, we showed for the first time that detergent-insoluble aggregates increase ~2.7-fold after MI relative to sham mice (unpublished data). Of the cardiac proteins enriched after experimental MI, ~9% coincided with proteins previously observed in aged and hypertensive heart aggregates [Figure 1]. Proteins previously implicated in protein homeostasis failure associated with neurodegenerative and/or CVDs, such as CAND1, SerpinH1, NEDD4, UCP1, Plectin-1, 14-3-3^[155]^, ApoE, Cardiac Phospholamban, and HSP-90^[156]^, were shared in common between MI, aging, and hypertension — suggesting that these conditions accrue protein aggregates due to defects in the same or similar pathways [Figure 1].

The overlap among proteins enriched in cardiac aggregates due to aging, hypertension (HT), and experimental myocardial infarction (MI) implicates novel targets for therapeutic intervention.

## THERAPEUTIC IMPLICATIONS OF IMPAIRED PROTEIN HOMEOSTASIS

**Figure 2.**
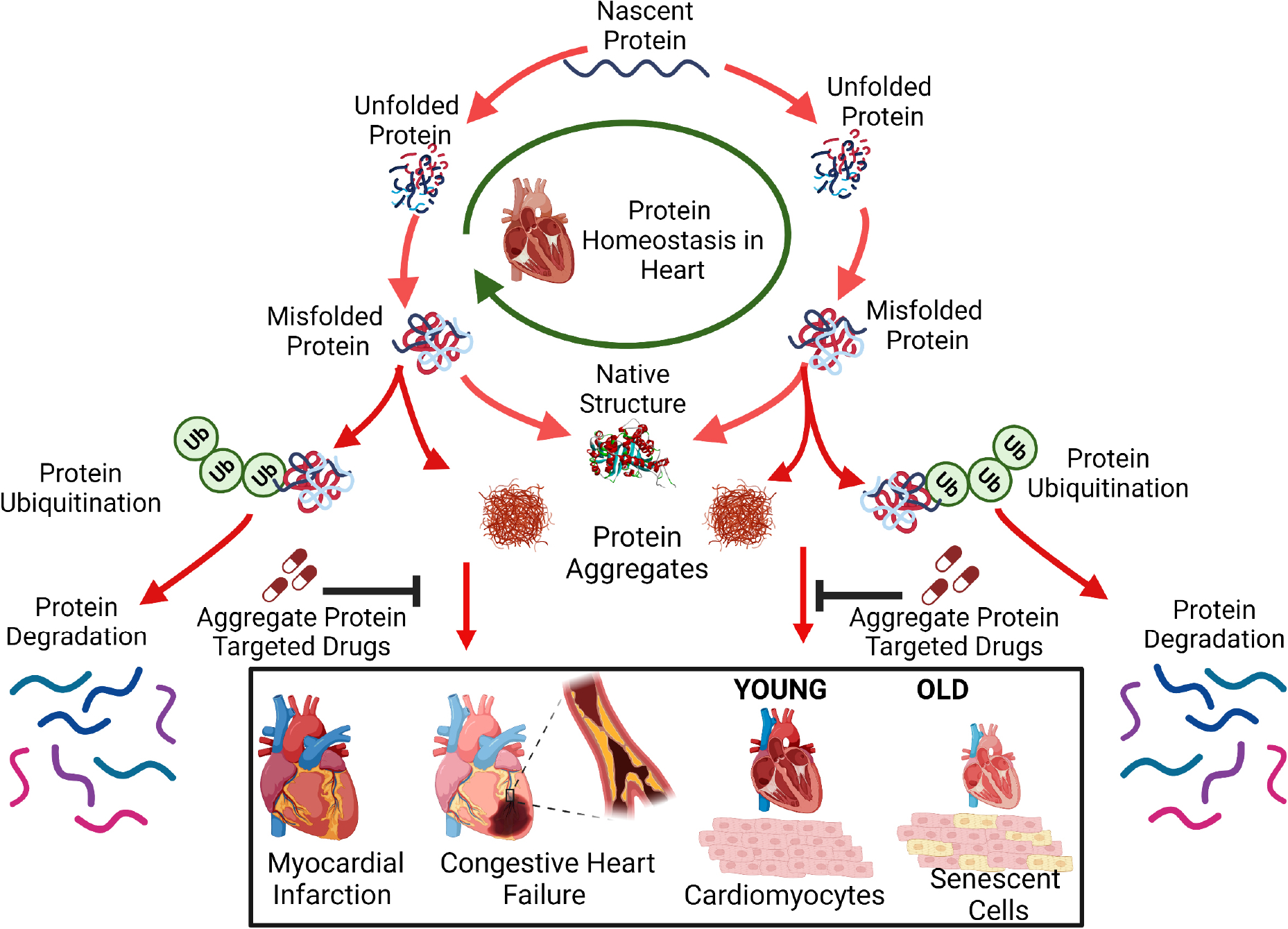
Drugs targeting cardiac-aggregate proteins can improve clearance of misfolded proteins and restore protein homeostasis compromised by cardiovascular disease (such as ischemia) and/or aging.

Proteins involved in UPS, autophagy, mitochondrial and ER stress, and those directly affected by hypoxia and oxidative stress, are regulated by known pathways and suggest novel therapeutic interventions to mitigate CVDs. These proteins can either be upregulated, silenced, or otherwise manipulated to restore or maintain proteostatic equilibrium [Figure 2]. Drugs can be screened and evaluated for affinity to these proteins by methods employed previously^[157–162]^, thus regulating their activity or interactions. Mitochondrial and ER proteins (e.g., PERK, IRE1, eiF2α, BiP, CHOP, GRP78, XBP1, ATF6, and ATF4) are known to be altered in diverse neurodegenerative diseases, and may be similarly aberrant in cardiac tissue, leading to protein aggregation and cardiomyopathy. In addition, autophagy regulators that include Atg4, Atg7, Atg3, and Atg10 have also been documented as reporters of CVD^[163–165]^.

Proteins may misfold during translation or subsequently due to oxidation, other damage, or excessive post-translational modification. Misfolded proteins may be refolded with the help of chaperones, but if they remain in a disordered state, they are vulnerable to aggregation. Individual proteins or complexes can be cleared by the ubiquitin-proteasome system, but if not, then they can only be eliminated via autophagy.

Additional proteins specific to myocardial ischemia and related conditions, as well as those shared with hypertension and aging, may be implicated as contributors to abnormal proteostasis. Understanding protein homeostasis pathways and how aggregated protein components contribute to aggregate accumulation and consequent functional decline are novel areas of cardiovascular research. Unconventional methods developed to combine proteomics and interactome analyses have allowed us to identify proteins and protein-protein interfaces that are especially influential for aggregation. These provide novel targets for the discovery of drugs that oppose aggregation [Figure 2] as described recently^[166]^.

Since dysfunctions of proteasomes and autophagy have been implicated in aging and age-related diseases, with consequences as diverse as those of Alzheimer’s disease and CVD, small molecules targeting influential aggregate proteins may also benefit those afflicted with a wide range of other age-associated conditions^[167,168]^. Targeting these aggregate-specific proteins with small molecules rescued aggregate-associated functional declines in AD models^[155,157,160,166,169]^. In view of the substantial overlaps between proteins sequestered into aggregates in aged, hypertensive and post-MI hearts, these aggregate-specific proteins and their interfaces (especially the shared protein sets) offer attractive drug targets to relieve protein aggregation and its associated physiological impairments. Therapies that improve cardiac function impaired by cardiac aging or by specific CVD events, especially myocardial ischemia, may target proteins defective in multiple pathologies, and thus serve as therapeutic approaches with the potential to ameliorate a wide range of age-related diseases.

## Data Availability

Not applicable.
